# Supplementary material for: Comparison of altruistic, egoistic, and scientific appeals in the recruitment of politicians to a survey panel
Source: PLoS One. 2026 May 28;21(5):e0349116. doi: 10.1371/journal.pone.0349116 (PMC13218533; doi:10.1371/journal.pone.0349116)
Supplement: S1 File — Online Appendix. (DOCX) [file pone.0349116.s001.docx]

**Online appendix**

**Appendix A**

**Appendix**

**Variables**

- recruited
  - yes
  - no
- treatment
  - Egoistic
  - Scientific
  - Altruistic
- gender
  - Man
  - Woman
- age_center: age of the person, centered around the mean
- agegrp4: age group of the person
  - 18-34
  - 35-49
  - 50-64
  - 65-
- lang (mother tongue)
  - Finnish
  - Swedish
  - Other
- municipality_type (Statistics Finland classification)
  - Urban
  - Suburban
  - Rural
- language_ratio (the language ratio in the municipality where the person live, Statistics Finland classification)
  - Finnish monolingual
  - Finnish majority bilingual
  - Swedish majority bilingual
- party_leftright (what type of political party does the person represent)
  - Left (SDP, VAS, VIHR)
  - Right (KOK, PS, KESK, RKP, KD, LIIK)
  - Other = NA

**Table A1.** Randomization check of the treatment groups by demographics

|  | **Recruited** | | | |  |
| --- | --- | --- | --- | --- | --- |
| **Variable** | **Egotistic N = 2,465** | **Scientific N = 2,466** | **Altruistic N = 2,466** | **Overall N = 7,397** | **p-value** |
| **Gender** |  |  |  |  | 0.7 |
| Man | 1,518 (62%) | 1,486 (60%) | 1,501 (61%) | 4,505 (61%) |  |
| Woman | 947 (38%) | 977 (40%) | 964 (39%) | 2,888 (39%) |  |
| **Age group** |  |  |  |  | 0.6 |
| 18-34 | 178 (7.3%) | 170 (7.0%) | 198 (8.1%) | 546 (7.5%) |  |
| 35-49 | 802 (33%) | 797 (33%) | 770 (31%) | 2,369 (32%) |  |
| 50-64 | 876 (36%) | 915 (38%) | 903 (37%) | 2,694 (37%) |  |
| 65- | 582 (24%) | 558 (23%) | 579 (24%) | 1,719 (23%) |  |
| **Language** |  |  |  |  | 0.8 |
| Finnish | 2,318 (94%) | 2,310 (94%) | 2,319 (94%) | 6,947 (94%) |  |
| Other | 147 (6.0%) | 156 (6.3%) | 147 (6.0%) | 450 (6.1%) |  |
| **Municipality type** |  |  |  |  | 0.6 |
| Urban | 788 (32%) | 770 (31%) | 741 (30%) | 2,299 (31%) |  |
| Suburban | 655 (27%) | 643 (26%) | 660 (27%) | 1,958 (26%) |  |
| Rural | 1,022 (41%) | 1,053 (43%) | 1,065 (43%) | 3,140 (42%) |  |
| **Municipality language ratio** |  |  |  |  | 0.7 |
| Finnish monolingual | 2,148 (87%) | 2,161 (88%) | 2,161 (88%) | 6,470 (87%) |  |
| Finnish majority bilingual | 215 (8.7%) | 194 (7.9%) | 193 (7.8%) | 602 (8.1%) |  |
| Swedish majority bilingual | 102 (4.1%) | 111 (4.5%) | 112 (4.5%) | 325 (4.4%) |  |
| **Political party classification** |  |  |  |  | 0.4 |
| Left | 686 (29%) | 646 (27%) | 647 (27%) | 1,979 (28%) |  |
| Right | 1,692 (71%) | 1,719 (73%) | 1,737 (73%) | 5,148 (72%) |  |
| n (%) | | | | | |
| Pearson's Chi-squared test | | | | | |

**Table A2.** Descriptives of the entire sample

|  | **Recruited** | | |
| --- | --- | --- | --- |
| **Variable** | **no N = 6,964** | **yes N = 433** | **Overall N = 7,397** |
| **Treatment group** |  |  |  |
| Egotistic | 2,321 (33%) | 144 (33%) | 2,465 (33%) |
| Scientific | 2,313 (33%) | 153 (35%) | 2,466 (33%) |
| Altruistic | 2,330 (33%) | 136 (31%) | 2,466 (33%) |
| **Gender** |  |  |  |
| Man | 4,276 (61%) | 229 (53%) | 4,505 (61%) |
| Woman | 2,685 (39%) | 203 (47%) | 2,888 (39%) |
| **Age group** |  |  |  |
| 18-34 | 529 (7.7%) | 17 (3.9%) | 546 (7.5%) |
| 35-49 | 2,268 (33%) | 101 (23%) | 2,369 (32%) |
| 50-64 | 2,533 (37%) | 161 (37%) | 2,694 (37%) |
| 65- | 1,565 (23%) | 154 (36%) | 1,719 (23%) |
| **Language** |  |  |  |
| Finnish | 6,556 (94%) | 391 (90%) | 6,947 (94%) |
| Other | 408 (5.9%) | 42 (9.7%) | 450 (6.1%) |
| **Municipality type** |  |  |  |
| Urban | 2,131 (31%) | 168 (39%) | 2,299 (31%) |
| Suburban | 1,850 (27%) | 108 (25%) | 1,958 (26%) |
| Rural | 2,983 (43%) | 157 (36%) | 3,140 (42%) |
| **Municipality language ratio** |  |  |  |
| Finnish monolingual | 6,129 (88%) | 341 (79%) | 6,470 (87%) |
| Finnish majority bilingual | 542 (7.8%) | 60 (14%) | 602 (8.1%) |
| Swedish majority bilingual | 293 (4.2%) | 32 (7.4%) | 325 (4.4%) |
| **Political party classification** |  |  |  |
| Left | 1,835 (27%) | 144 (36%) | 1,979 (28%) |
| Right | 4,890 (73%) | 258 (64%) | 5,148 (72%) |

**Table A3.** Odds ratios of recruited

| **Characteristic** | **OR** | **95% CI** | **p-value** |
| --- | --- | --- | --- |
| Treatment |  |  |  |
| Egotistic / Scientific | 0.95 | 0.71, 1.28 | 0.915 |
| Egotistic / Altruistic | 1.08 | 0.80, 1.47 | 0.807 |
| Scientific / Altruistic | 1.14 | 0.85, 1.54 | 0.56 |
| Gender |  |  |  |
| Man / Woman | 0.64*** | 0.52, 0.79 | **<0.001** |
| Age centered | 1.03*** | 1.02, 1.04 | **<0.001** |
| Language |  |  |  |
| Finnish / Other | 0.78 | 0.49, 1.26 | 0.311 |
| Municipality type |  |  |  |
| Urban / Suburban | 1.2 | 0.87, 1.67 | 0.373 |
| Urban / Rural | 1.37* | 1.01, 1.85 | **0.042** |
| Suburban / Rural | 1.13 | 0.83, 1.56 | 0.623 |
| Municipality language ratio |  |  |  |
| Finnish monolingual / Finnish majority bilingual | 0.58** | 0.39, 0.87 | **0.004** |
| Finnish monolingual / Swedish majority bilingual | 0.52* | 0.27, 0.98 | **0.043** |
| Finnish majority bilingual / Swedish majority bilingual | 0.89 | 0.46, 1.71 | 0.907 |
| Party type |  |  |  |
| Left / Right | 1.31* | 1.05, 1.63 | **0.017** |
| *p<0.05; **p<0.01; ***p<0.001 | | | |
| Abbreviations: CI = Confidence Interval, OR = Odds Ratio | | | |
| Note: Bonferroni correction used to account for the multiple corrections problem | | | |


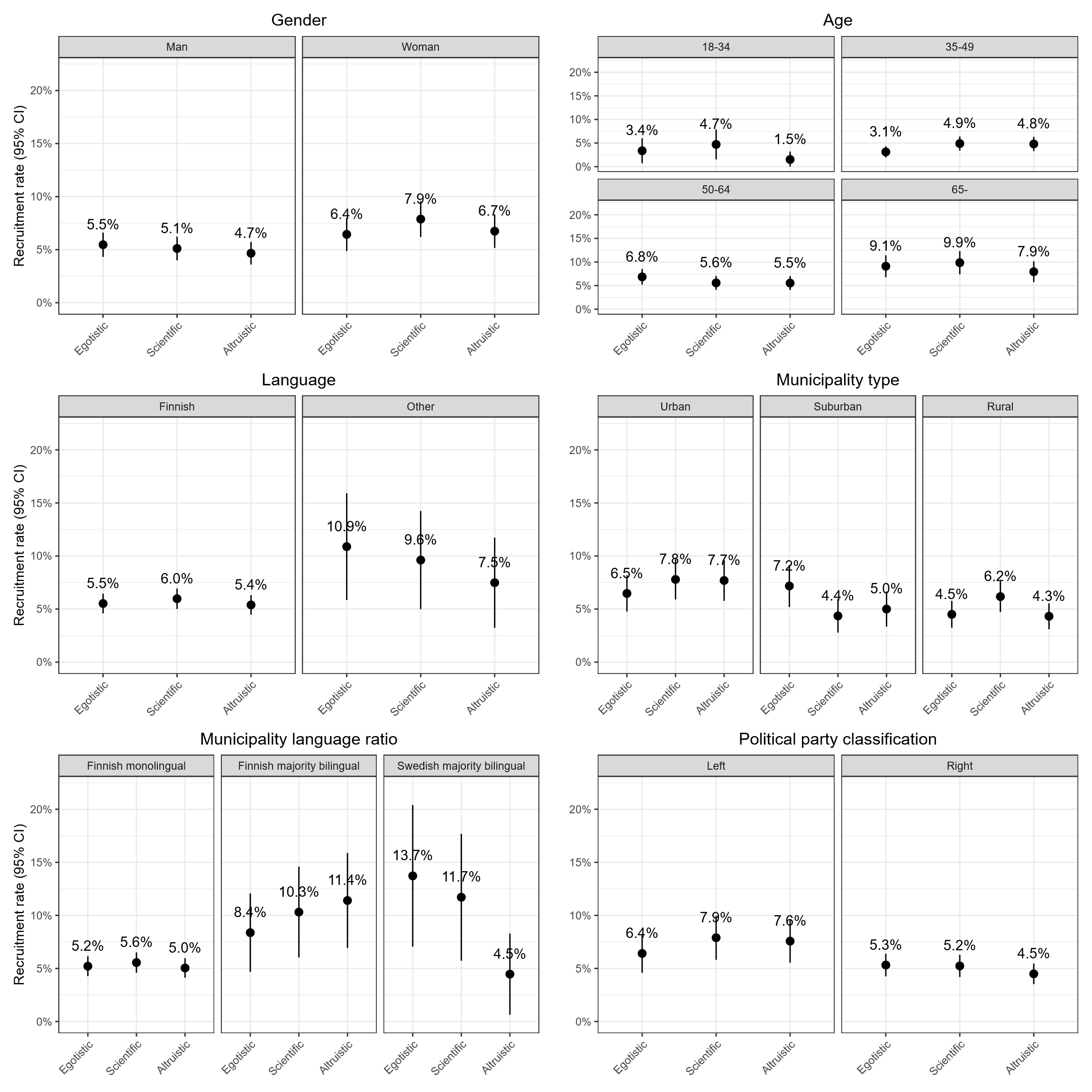


**Figure A1.** Groupwise recruitment rates and 95% confidence intervals by demographics and treatments.

**Table A4.** Groupwise recruitment rates by demographics and treatments.

|  | **Egotistic** | **Scientific** | **Altruistic** | **Overall** | **p-value*** |
| --- | --- | --- | --- | --- | --- |
|  |  |  |  |  |  |
| **Overall** | 5.8% | 6.2% | 5.5% | 5.9% | 0.6 |
|  |  |  |  |  |  |
| **Gender** |  |  |  |  |  |
| Man | 5.5% | 5.1% | 4.7% | 5.1% | 0.6 |
| Woman | 6.4% | 7.9% | 6.7% | 7.0% | 0.4 |
|  |  |  |  |  |  |
| **Age** |  |  |  |  |  |
| 18-34 | 3.4% | 4.7% | 1.5% | 3.1% | 0.2 |
| 35-49 | 3.1% | 4.9% | 4.8% | 4.3% | 0.14 |
| 50-64 | 6.8% | 5.6% | 5.5% | 6.0% | 0.4 |
| 65- | 9.1% | 9.9% | 7.9% | 9.0% | 0.5 |
|  |  |  |  |  |  |
| **Language** |  |  |  |  |  |
| Finnish | 5.5% | 6.0% | 5.4% | 5.6% | 0.7 |
| Other | 10.9% | 9.6% | 7.5% | 9.3% | 0.6 |
|  |  |  |  |  |  |
| **Municipality type** |  |  |  |  |  |
| Urban | 6.5% | 7.8% | 7.7% | 7.3% | 0.5 |
| Suburban | 7.2% | 4.4% | 5.0% | 5.5% | 0.065 |
| Rural | 4.5% | 6.2% | 4.3% | 5.0% | 0.1 |
|  |  |  |  |  |  |
| **Municipality language ratio** |  |  |  |  |  |
| Finnish monolingual | 5.2% | 5.6% | 5.0% | 5.3% | 0.7 |
| Finnish majority bilingual | 8.4% | 10.3% | 11.4% | 10.0% | 0.6 |
| Swedish majority bilingual | 13.7% | 11.7% | 4.5% | 9.8% | 0.055 |
|  |  |  |  |  |  |
| **Political party classification** |  |  |  |  |  |
| Left | 6.4% | 7.9% | 7.6% | 7.3% | 0.5 |
| Right | 5.3% | 5.2% | 4.5% | 5.0% | 0.5 |
|  |  |  |  |  |  |
| *Pearson's Chi-squared test |  |  |  |  |  |

**Model fit**

Models:

- m1: recruited ~ treatment
- m2: recruited ~ treatment + gender + age_center + lang
- m3: recruited ~ treatment + gender + age_center + lang + municipality_type + language_ratio
- m4: recruited ~ treatment + gender + age_center + lang + municipality_type + language_ratio + party_leftright

**Table A5.** Model performance

| Name | Model | AIC | AIC_wt | AICc | AICc_wt | BIC | BIC_wt | R2_Tjur | RMSE | Sigma | Log_loss | Score_log | Score_spherical | PCP |
| --- | --- | --- | --- | --- | --- | --- | --- | --- | --- | --- | --- | --- | --- | --- |
| m1 | glm | 3303 | 2,4767E-69 | 3303 | 2,5194E-69 | 3324 | 1,9262E-57 | 0,000 | 0,235 | 1 | 0,223 | -25,928 | 0,003 | 0,890 |
| m2 | glm | 3214 | 5,401E-50 | 3214 | 5,4716E-50 | 3255 | 1,3645E-42 | 0,012 | 0,234 | 1 | 0,218 | -26,218 | 0,001 | 0,890 |
| m3 | glm | 3196 | 4,8788E-46 | 3196 | 4,8968E-46 | 3265 | 1,2536E-44 | 0,017 | 0,234 | 1 | 0,217 | -26,283 | 0,001 | 0,891 |
| m4 | glm | 2987 | 1 | 2987 | 1 | 3062 | 1 | 0,018 | 0,229 | 1 | 0,210 | -23,486 | 0,001 | 0,895 |

**
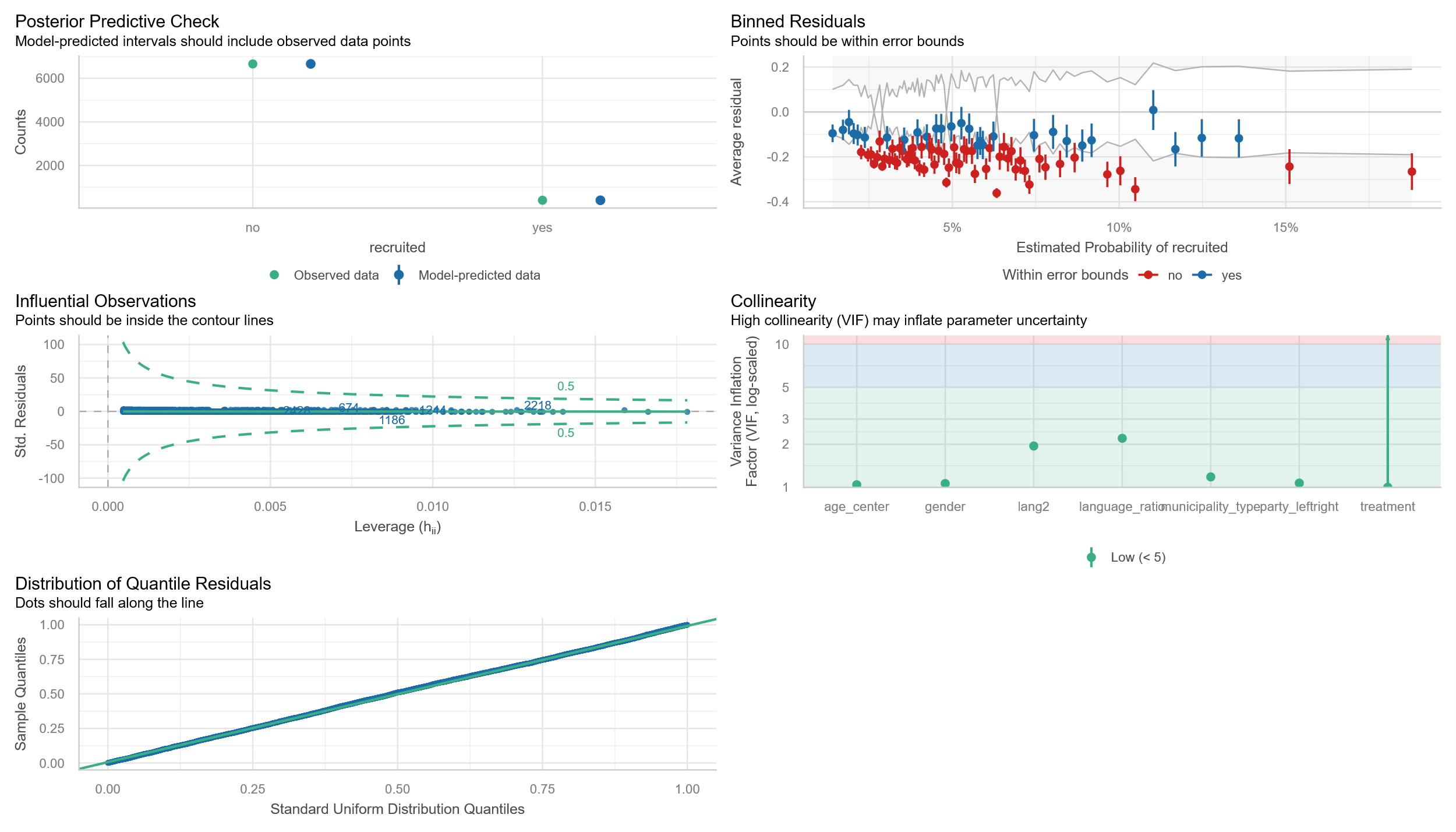
**

**Figure A2.** Performance of model m4

**Table** **A6**. Predicting recruitment

|  | | | | |
| --- | --- | --- | --- | --- |
|  | Dependent variable: | | | |
|  |  | | | |
|  | Recruited | | | |
|  | (1) | (2) | (3) | (4) |
|  | | | | |
| Treatment: Scientific | 0.064 | 0.055 | 0.059 | 0.050 |
|  | (0.120) | (0.121) | (0.121) | (0.126) |
|  |  |  |  |  |
| Treatment: Altruistic | -0.061 | -0.086 | -0.082 | -0.080 |
|  | (0.123) | (0.124) | (0.124) | (0.129) |
|  |  |  |  |  |
| Gender: Woman |  | 0.496^***^ | 0.465^***^ | 0.445^***^ |
|  |  | (0.102) | (0.102) | (0.107) |
|  |  |  |  |  |
| Age centered |  | 0.031^***^ | 0.031^***^ | 0.032^***^ |
|  |  | (0.004) | (0.004) | (0.004) |
|  |  |  |  |  |
| Language: Other |  | 0.604^***^ | 0.099 | 0.244 |
|  |  | (0.172) | (0.236) | (0.241) |
|  |  |  |  |  |
| Municipality type: Suburban |  |  | -0.224 | -0.186 |
|  |  |  | (0.134) | (0.139) |
|  |  |  |  |  |
| Municipality type: Rural |  |  | -0.352^**^ | -0.311^*^ |
|  |  |  | (0.123) | (0.129) |
|  |  |  |  |  |
| Municipality language ratio: Finnish majority bilingual |  |  | 0.533^**^ | 0.545^**^ |
|  |  |  | (0.167) | (0.171) |
|  |  |  |  |  |
| Municipality language ratio: Swedish majority bilingual |  |  | 0.707^**^ | 0.662^*^ |
|  |  |  | (0.265) | (0.276) |
|  |  |  |  |  |
| Party type: Right |  |  |  | -0.269^*^ |
|  |  |  |  | (0.113) |
|  |  |  |  |  |
| Constant | -2.780^***^ | -3.079^***^ | -2.930^***^ | -2.820^***^ |
|  | (0.086) | (0.102) | (0.127) | (0.148) |
|  |  |  |  |  |
|  | | | | |
| Observations | 7,397 | 7,327 | 7,327 | 7,061 |
| Log Likelihood | -1,648.434 | -1,600.905 | -1,587.796 | -1,482.462 |
| Akaike Inf. Crit. | 3,302.868 | 3,213.810 | 3,195.593 | 2,986.925 |
|  | | | | |
| Note: | ^* p < 0.05, ** p < 0.01, *** p < 0.001^ | | | |

**Table A7.** Robustness test comparing model 4 MLE logistic regression with a Firth’s logistic regression.

|  | Logit (MLE) | Logit (Firth) |
| --- | --- | --- |
| Scientific incentive | 0.050 | 0.050 |
|  | (0.126) | (0.125) |
| Altruistic incentive | -0.080 | -0.080 |
|  | (0.129) | (0.128) |
| Woman | 0.445*** | 0.444*** |
|  | (0.107) | (0.107) |
| Age (centered) | 0.032*** | 0.032*** |
|  | (0.004) | (0.004) |
| Other language | 0.244 | 0.251 |
|  | (0.241) | (0.238) |
| Suburban municipality | -0.186 | -0.184 |
|  | (0.139) | (0.138) |
| Rural municipality | -0.311* | -0.311* |
|  | (0.129) | (0.128) |
| Finnish-majority bilingual area | 0.545** | 0.551** |
|  | (0.171) | (0.170) |
| Swedish-majority bilingual area | 0.662* | 0.672* |
|  | (0.276) | (0.272) |
| Right-leaning | -0.269* | -0.271* |
|  | (0.113) | (0.112) |
| Num.Obs. | 7061 | 7061 |
| R2 |  | 0.018 |
| AIC | 2986.9 |  |
| BIC | 3062.4 |  |
| Log.Lik. | -1482.462 |  |
| RMSE | 0.23 | 1.03 |
| * p < 0.05, ** p < 0.01, *** p < 0.001 | | |

**Table A8.** Analysis of Deviance for model 4

|  | LR Chisq | Df | Pr(>Chisq) |
| --- | --- | --- | --- |
| Treatment | 1.069 | 2 | 0.586 |
| Gender | 17.026 | 1 | 0.000 |
| Age centered | 58.809 | 1 | 0.000 |
| Language | 1.006 | 1 | 0.316 |
| Municipality type | 5.804 | 2 | 0.055 |
| Municipality language ratio | 11.666 | 2 | 0.003 |
| Party type | 5.605 | 1 | 0.018 |

**Table A9.** Predicting recruitment with interactions

|  | | | | | | |
| --- | --- | --- | --- | --- | --- | --- |
|  | Dependent variable: | | | | | |
|  |  | | | | | |
|  | Recruited | | | | | |
|  | (1) | (2) | (3) | (4) | (5) | (6) |
|  | | | | | | |
| Treatment: Scientific | -0.058 | 0.061 | 0.080 | 0.176 | 0.081 | 0.216 |
|  | (0.170) | (0.132) | (0.132) | (0.203) | (0.141) | (0.214) |
|  |  |  |  |  |  |  |
| Treatment: Altruistic | -0.153 | -0.055 | -0.033 | 0.171 | -0.024 | 0.157 |
|  | (0.173) | (0.134) | (0.135) | (0.204) | (0.144) | (0.216) |
|  |  |  |  |  |  |  |
| Gender: Woman | 0.228 |  |  |  |  |  |
|  | (0.180) |  |  |  |  |  |
|  |  |  |  |  |  |  |
| Treatment: Scientific x Gender: Woman | 0.240 |  |  |  |  |  |
|  | (0.250) |  |  |  |  |  |
|  |  |  |  |  |  |  |
| Treatment: Altruistic x Gender: Woman | 0.168 |  |  |  |  |  |
|  | (0.257) |  |  |  |  |  |
|  |  |  |  |  |  |  |
| Age centered |  | 0.030^***^ |  |  |  |  |
|  |  | (0.007) |  |  |  |  |
|  |  |  |  |  |  |  |
| Treatment: Scientific x Age centered |  | -0.001 |  |  |  |  |
|  |  | (0.010) |  |  |  |  |
|  |  |  |  |  |  |  |
| Treatment: Altruistic x Age centered |  | -0.005 |  |  |  |  |
|  |  | (0.010) |  |  |  |  |
|  |  |  |  |  |  |  |
| Language: Other |  |  | 0.819^**^ |  |  |  |
|  |  |  | (0.282) |  |  |  |
|  |  |  |  |  |  |  |
| Treatment: Scientific x Language: Other |  |  | -0.243 |  |  |  |
|  |  |  | (0.402) |  |  |  |
|  |  |  |  |  |  |  |
| Treatment: Altruistic x Language: Other |  |  | -0.419 |  |  |  |
|  |  |  | (0.432) |  |  |  |
|  |  |  |  |  |  |  |
| Municipality type: Suburban |  |  |  | 0.131 |  |  |
|  |  |  |  | (0.214) |  |  |
|  |  |  |  |  |  |  |
| Municipality type: Rural |  |  |  | -0.445^*^ |  |  |
|  |  |  |  | (0.219) |  |  |
|  |  |  |  |  |  |  |
| Treatment: Scientific x Municipality type: Suburban |  |  |  | -0.733^*^ |  |  |
|  |  |  |  | (0.324) |  |  |
|  |  |  |  |  |  |  |
| Treatment: Altruistic x Municipality type: Suburban |  |  |  | -0.625^*^ |  |  |
|  |  |  |  | (0.317) |  |  |
|  |  |  |  |  |  |  |
| Treatment: Scientific x Municipality type: Rural |  |  |  | 0.203 |  |  |
|  |  |  |  | (0.292) |  |  |
|  |  |  |  |  |  |  |
| Treatment: Altruistic x Municipality type: Rural |  |  |  | -0.172 |  |  |
|  |  |  |  | (0.305) |  |  |
|  |  |  |  |  |  |  |
| Municipality language ratio: Finnish majority bilingual |  |  |  |  | 0.587^*^ |  |
|  |  |  |  |  | (0.267) |  |
|  |  |  |  |  |  |  |
| Municipality language ratio: Swedish majority bilingual |  |  |  |  | 1.219^***^ |  |
|  |  |  |  |  | (0.307) |  |
|  |  |  |  |  |  |  |
| Treatment: Scientific x Municipality language ratio: Finnish majority bilingual |  |  |  |  | 0.141 |  |
|  |  |  |  |  | (0.370) |  |
|  |  |  |  |  |  |  |
| Treatment: Altruistic x Municipality language ratio: Finnish majority bilingual |  |  |  |  | 0.281 |  |
|  |  |  |  |  | (0.371) |  |
|  |  |  |  |  |  |  |
| Treatment: Scientific x Municipality language ratio: Swedish majority bilingual |  |  |  |  | -0.507 |  |
|  |  |  |  |  | (0.453) |  |
|  |  |  |  |  |  |  |
| Treatment: Altruistic x Municipality language ratio: Swedish majority bilingual |  |  |  |  | -1.281^*^ |  |
|  |  |  |  |  | (0.561) |  |
|  |  |  |  |  |  |  |
| Party type: Right |  |  |  |  |  | -0.202 |
|  |  |  |  |  |  | (0.190) |
|  |  |  |  |  |  |  |
| Treatment: Scientific x Party type: Right |  |  |  |  |  | -0.233 |
|  |  |  |  |  |  | (0.263) |
|  |  |  |  |  |  |  |
| Treatment: Altruistic x Party type: Right |  |  |  |  |  | -0.342 |
|  |  |  |  |  |  | (0.268) |
|  |  |  |  |  |  |  |
| Constant | -2.898^***^ | -2.870^***^ | -2.875^***^ | -2.687^***^ | -2.949^***^ | -2.666^***^ |
|  | (0.118) | (0.094) | (0.095) | (0.148) | (0.102) | (0.156) |
|  |  |  |  |  |  |  |
|  | | | | | | |
| Observations | 7,061 | 7,061 | 7,061 | 7,061 | 7,061 | 7,061 |
| Log Likelihood | -1,532.378 | -1,515.564 | -1,533.042 | -1,527.441 | -1,521.299 | -1,531.847 |
| Akaike Inf. Crit. | 3,076.757 | 3,043.129 | 3,078.083 | 3,072.882 | 3,060.598 | 3,075.694 |
|  | | | | | | |
| Note: | ^*p<0.05; **p<0.01; ***p<0.001^ | | | | | |


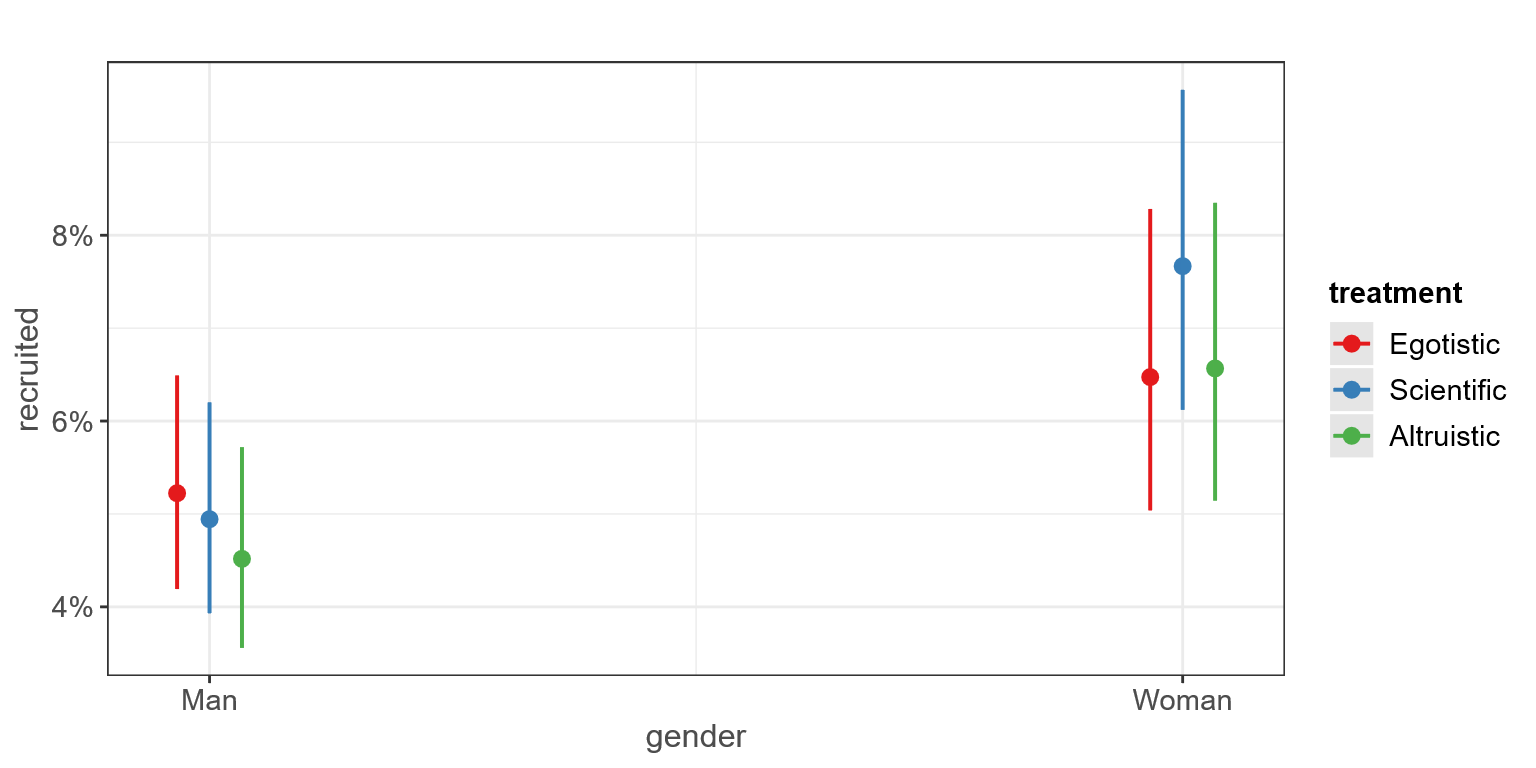


**Figure A3**. Interaction gender and treatment

^
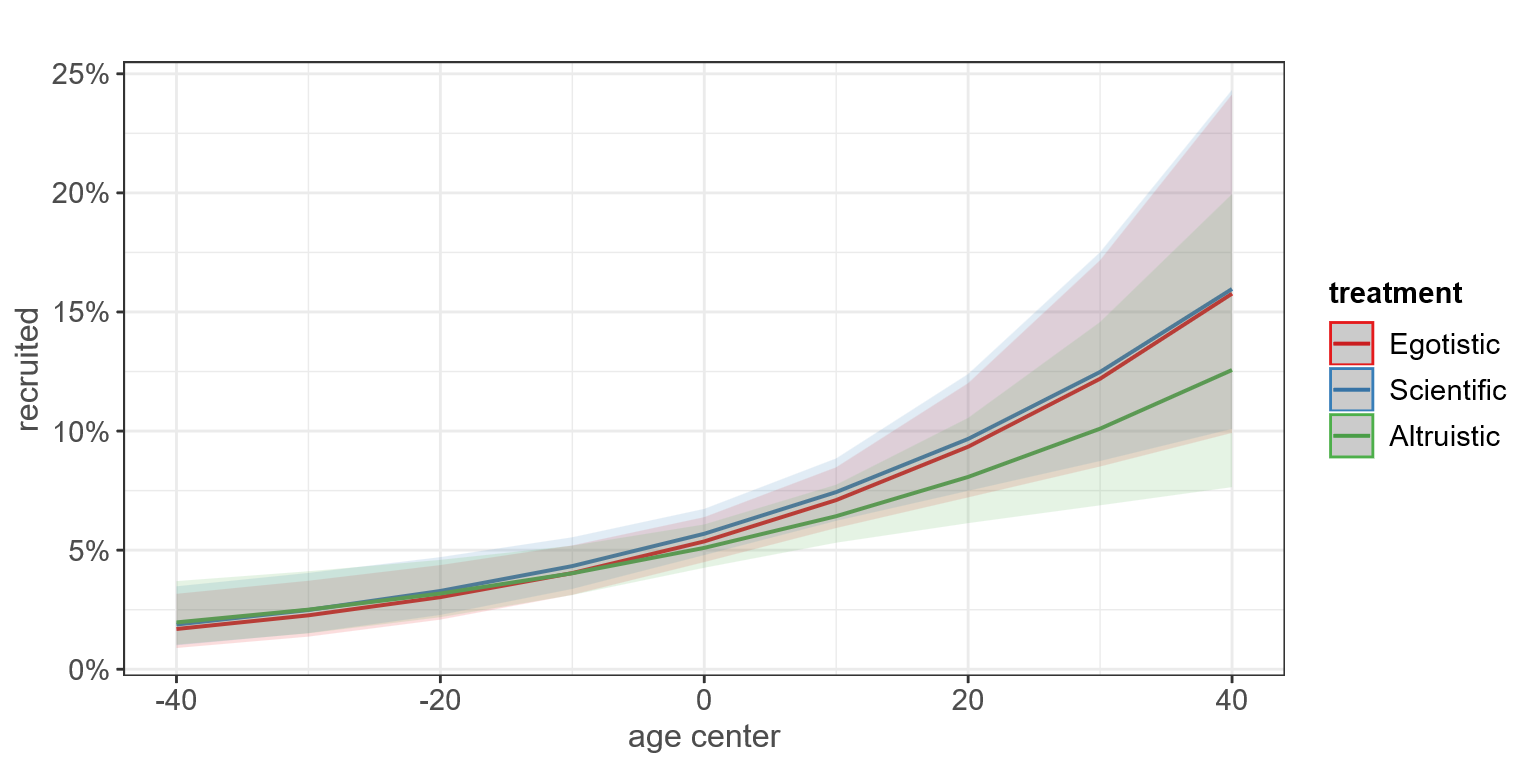
^

**Figure A4**. Interaction age and treatment


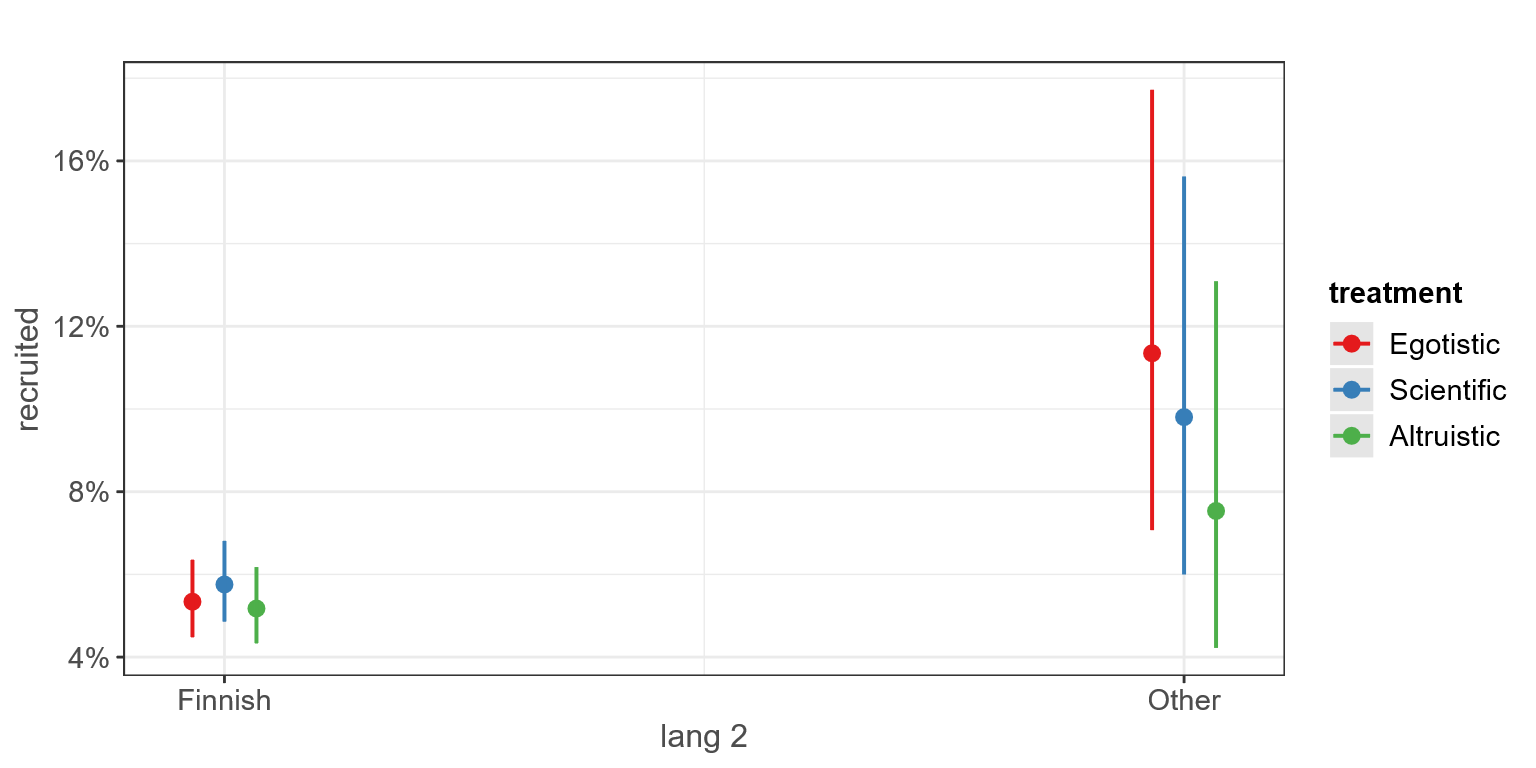


**Figure A5**. Interaction language and treatment

**Appendix B**

**Model fit**

Models:

- m1: recruited ~ treatment
- m2: recruited ~ treatment + gender + age_center + lang
- m3: recruited ~ treatment + gender + age_center + lang + municipality_type + language_ratio
- m4: recruited ~ treatment + gender + age_center + lang + municipality_type + language_ratio + party_leftright
- m5: recruited ~ treatment + gender + age_center + lang + municipality_type + language_ratio + party_big

**Table B2.** Model performance

Name | Model | AIC (weights) | AICc (weights) | BIC (weights) | Tjur's R2 | RMSE | Sigma | Log_loss | Score_log | Score_spherical | PCP

--------------------------------------------------------------------------------------------------------------------------------------------

m1 | glm | 3302.9 (<.001) | 3302.9 (<.001) | 3323.6 (<.001) | 1.439e-04 | 0.235 | 1.000 | 0.223 | -25.928 | 0.003 | 0.890

m2 | glm | 3215.5 (<.001) | 3215.6 (<.001) | 3263.8 (<.001) | 0.012 | 0.234 | 1.000 | 0.218 | -26.220 | 9.594e-04 | 0.890

m3 | glm | 3197.6 (<.001) | 3197.6 (<.001) | 3273.5 (<.001) | 0.017 | 0.234 | 1.000 | 0.217 | -26.283 | 9.083e-04 | 0.891

m4 | glm | 2988.8 (>.999) | 2988.9 (>.999) | 3071.2 (>.999) | 0.018 | 0.229 | 1.000 | 0.210 | -23.486 | 0.001 | 0.895

m5 | glm | 3191.0 (<.001) | 3191.1 (<.001) | 3294.5 (<.001) | 0.019 | 0.233 | 1.000 | 0.216 | -26.312 | 0.001 | 0.891

**
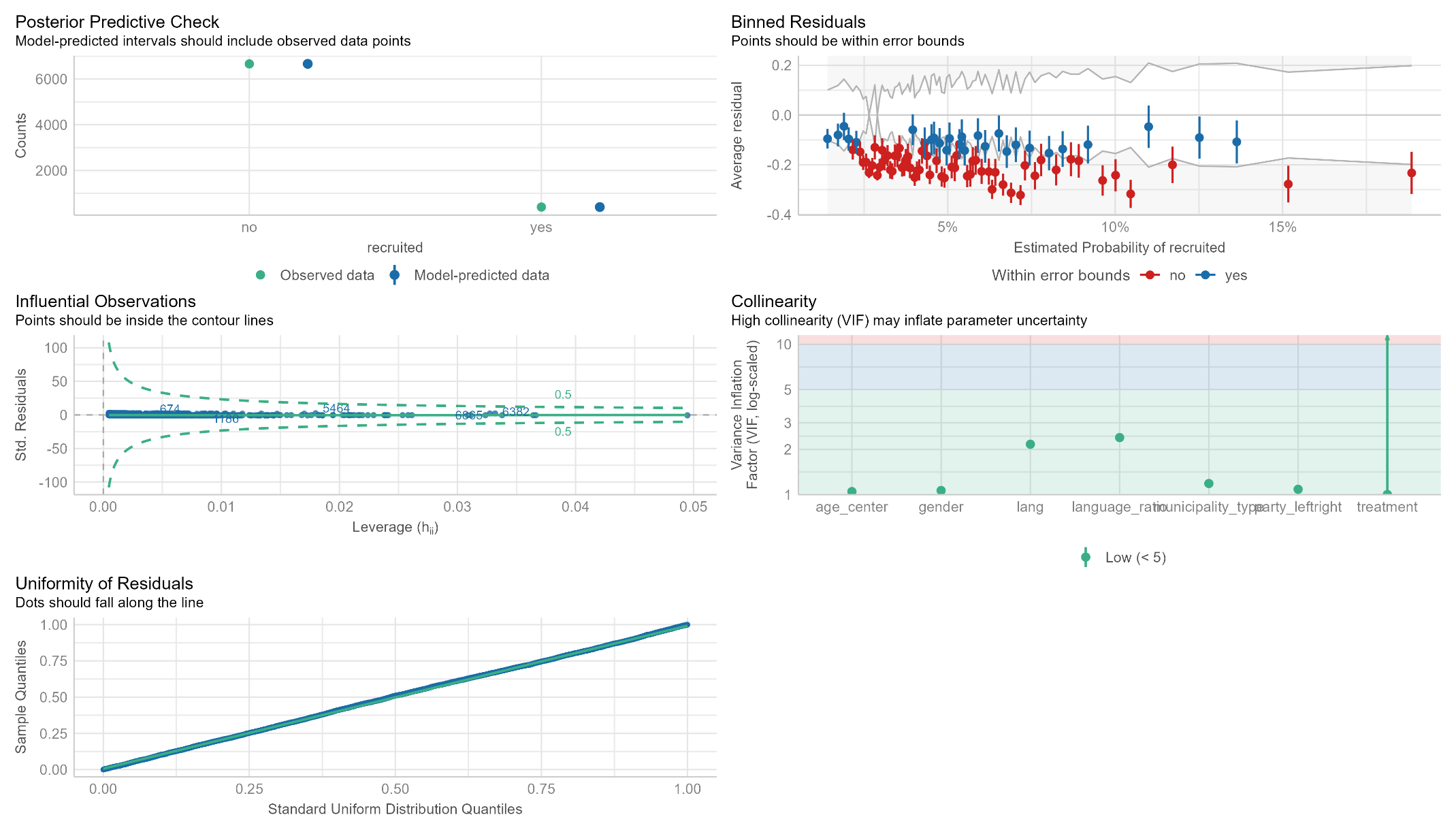
**

**Figure B1.** Performance of model m4

**Table B3.** Predicting recruitment

|  | | | | | |
| --- | --- | --- | --- | --- | --- |
|  | | | | | |
|  | *Dependent variable:* | | | | |
|  |  | | | | |
|  | recruited | | | | |
|  | (1) | (2) | (3) | (4) | (5) |
|  | | | | | |
| treatmentScientific | 0.064 | 0.054 | 0.059 | 0.050 | 0.055 |
|  | (0.120) | (0.121) | (0.121) | (0.126) | (0.121) |
|  |  |  |  |  |  |
| treatmentAltruistic | -0.066 | -0.086 | -0.082 | -0.080 | -0.081 |
|  | (0.123) | (0.124) | (0.124) | (0.129) | (0.125) |
|  |  |  |  |  |  |
| genderWoman |  | 0.495^***^ | 0.465^***^ | 0.444^***^ | 0.420^***^ |
|  |  | (0.102) | (0.102) | (0.107) | (0.105) |
|  |  |  |  |  |  |
| age_center |  | 0.031^***^ | 0.031^***^ | 0.032^***^ | 0.031^***^ |
|  |  | (0.004) | (0.004) | (0.004) | (0.004) |
|  |  |  |  |  |  |
| langSwedish |  | 0.630^***^ | 0.103 | 0.277 | -0.106 |
|  |  | (0.178) | (0.253) | (0.261) | (0.261) |
|  |  |  |  |  |  |
| langOther |  | 0.320 | 0.079 | 0.069 | -0.017 |
|  |  | (0.601) | (0.604) | (0.607) | (0.605) |
|  |  |  |  |  |  |
| municipality_typeSuburban |  |  | -0.224 | -0.188 | -0.175 |
|  |  |  | (0.134) | (0.139) | (0.135) |
|  |  |  |  |  |  |
| municipality_typeRural |  |  | -0.353^**^ | -0.313^*^ | -0.280^*^ |
|  |  |  | (0.123) | (0.129) | (0.126) |
|  |  |  |  |  |  |
| language_ratioFinnish majority bilingual |  |  | 0.532^**^ | 0.539^**^ | 0.490^**^ |
|  |  |  | (0.168) | (0.172) | (0.169) |
|  |  |  |  |  |  |
| language_ratioSwedish majority bilingual |  |  | 0.704^*^ | 0.636^*^ | 0.583^*^ |
|  |  |  | (0.274) | (0.287) | (0.274) |
|  |  |  |  |  |  |
| party_leftrightRight |  |  |  | -0.273^*^ |  |
|  |  |  |  | (0.113) |  |
|  |  |  |  |  |  |
| party_bigPS |  |  |  |  | -0.078 |
|  |  |  |  |  | (0.191) |
|  |  |  |  |  |  |
| party_bigSDP |  |  |  |  | 0.132 |
|  |  |  |  |  | (0.168) |
|  |  |  |  |  |  |
| party_bigKESK |  |  |  |  | -0.202 |
|  |  |  |  |  | (0.170) |
|  |  |  |  |  |  |
| party_bigOther |  |  |  |  | 0.358^*^ |
|  |  |  |  |  | (0.161) |
|  |  |  |  |  |  |
| Constant | -2.768^***^ | -3.078^***^ | -2.930^***^ | -2.814^***^ | -2.983^***^ |
|  | (0.086) | (0.102) | (0.128) | (0.149) | (0.168) |
|  |  |  |  |  |  |
|  | | | | | |
| Observations | 7,328 | 7,327 | 7,327 | 7,061 | 7,327 |
| Log Likelihood | -1,644.214 | -1,600.772 | -1,587.796 | -1,482.409 | -1,580.525 |
| Akaike Inf. Crit. | 3,294.428 | 3,215.544 | 3,197.592 | 2,988.819 | 3,191.049 |
|  | | | | | |
| *Note:* | *p<0.05; **p<0.01; ***p<0.001 | | | | |

**Table B4.** Predicting recruitment with interactions

|  | | | | | | | |
| --- | --- | --- | --- | --- | --- | --- | --- |
|  | | | | | | | |
|  | *Dependent variable:* | | | | | | |
|  |  | | | | | | |
|  | recruited | | | | | | |
|  | (1) | (2) | (3) | (4) | (5) | (6) | (7) |
|  | | | | | | | |
| treatmentScientific | -0.071 | 0.074 | 0.083 | 0.200 | 0.067 | 0.224 | 0.220 |
|  | (0.163) | (0.126) | (0.126) | (0.198) | (0.135) | (0.213) | (0.299) |
|  |  |  |  |  |  |  |  |
| treatmentAltruistic | -0.168 | -0.045 | -0.026 | 0.186 | -0.035 | 0.179 | 0.062 |
|  | (0.167) | (0.129) | (0.129) | (0.200) | (0.138) | (0.215) | (0.313) |
|  |  |  |  |  |  |  |  |
| genderWoman | 0.174 |  |  |  |  |  |  |
|  | (0.174) |  |  |  |  |  |  |
|  |  |  |  |  |  |  |  |
| treatmentScientific:genderWoman | 0.288 |  |  |  |  |  |  |
|  | (0.241) |  |  |  |  |  |  |
|  |  |  |  |  |  |  |  |
| treatmentAltruistic:genderWoman | 0.217 |  |  |  |  |  |  |
|  | (0.248) |  |  |  |  |  |  |
|  |  |  |  |  |  |  |  |
| age_center |  | 0.030^***^ |  |  |  |  |  |
|  |  | (0.007) |  |  |  |  |  |
|  |  |  |  |  |  |  |  |
| treatmentScientific:age_center |  | -0.002 |  |  |  |  |  |
|  |  | (0.010) |  |  |  |  |  |
|  |  |  |  |  |  |  |  |
| treatmentAltruistic:age_center |  | -0.006 |  |  |  |  |  |
|  |  | (0.010) |  |  |  |  |  |
|  |  |  |  |  |  |  |  |
| langSwedish |  |  | 0.903^**^ |  |  |  |  |
|  |  |  | (0.282) |  |  |  |  |
|  |  |  |  |  |  |  |  |
| langOther |  |  | -12.726 |  |  |  |  |
|  |  |  | (325.437) |  |  |  |  |
|  |  |  |  |  |  |  |  |
| treatmentScientific:langSwedish |  |  | -0.283 |  |  |  |  |
|  |  |  | (0.403) |  |  |  |  |
|  |  |  |  |  |  |  |  |
| treatmentAltruistic:langSwedish |  |  | -0.770 |  |  |  |  |
|  |  |  | (0.470) |  |  |  |  |
|  |  |  |  |  |  |  |  |
| treatmentScientific:langOther |  |  | -0.083 |  |  |  |  |
|  |  |  | (507.157) |  |  |  |  |
|  |  |  |  |  |  |  |  |
| treatmentAltruistic:langOther |  |  | 14.125 |  |  |  |  |
|  |  |  | (325.437) |  |  |  |  |
|  |  |  |  |  |  |  |  |
| municipality_typeSuburban |  |  |  | 0.111 |  |  |  |
|  |  |  |  | (0.209) |  |  |  |
|  |  |  |  |  |  |  |  |
| municipality_typeRural |  |  |  | -0.384 |  |  |  |
|  |  |  |  | (0.209) |  |  |  |
|  |  |  |  |  |  |  |  |
| treatmentScientific:municipality_typeSuburban |  |  |  | -0.729^*^ |  |  |  |
|  |  |  |  | (0.315) |  |  |  |
|  |  |  |  |  |  |  |  |
| treatmentAltruistic:municipality_typeSuburban |  |  |  | -0.570 |  |  |  |
|  |  |  |  | (0.308) |  |  |  |
|  |  |  |  |  |  |  |  |
| treatmentScientific:municipality_typeRural |  |  |  | 0.134 |  |  |  |
|  |  |  |  | (0.280) |  |  |  |
|  |  |  |  |  |  |  |  |
| treatmentAltruistic:municipality_typeRural |  |  |  | -0.229 |  |  |  |
|  |  |  |  | (0.292) |  |  |  |
|  |  |  |  |  |  |  |  |
| language_ratioFinnish majority bilingual |  |  |  |  | 0.507 |  |  |
|  |  |  |  |  | (0.265) |  |  |
|  |  |  |  |  |  |  |  |
| language_ratioSwedish majority bilingual |  |  |  |  | 1.062^***^ |  |  |
|  |  |  |  |  | (0.304) |  |  |
|  |  |  |  |  |  |  |  |
| treatmentScientific:language_ratioFinnish majority bilingual |  |  |  |  | 0.163 |  |  |
|  |  |  |  |  | (0.367) |  |  |
|  |  |  |  |  |  |  |  |
| treatmentAltruistic:language_ratioFinnish majority bilingual |  |  |  |  | 0.377 |  |  |
|  |  |  |  |  | (0.362) |  |  |
|  |  |  |  |  |  |  |  |
| treatmentScientific:language_ratioSwedish majority bilingual |  |  |  |  | -0.248 |  |  |
|  |  |  |  |  | (0.434) |  |  |
|  |  |  |  |  |  |  |  |
| treatmentAltruistic:language_ratioSwedish majority bilingual |  |  |  |  | -1.190^*^ |  |  |
|  |  |  |  |  | (0.558) |  |  |
|  |  |  |  |  |  |  |  |
| party_leftrightRight |  |  |  |  |  | -0.199 |  |
|  |  |  |  |  |  | (0.190) |  |
|  |  |  |  |  |  |  |  |
| treatmentScientific:party_leftrightRight |  |  |  |  |  | -0.240 |  |
|  |  |  |  |  |  | (0.263) |  |
|  |  |  |  |  |  |  |  |
| treatmentAltruistic:party_leftrightRight |  |  |  |  |  | -0.357 |  |
|  |  |  |  |  |  | (0.267) |  |
|  |  |  |  |  |  |  |  |
| party_bigPS |  |  |  |  |  |  | -0.110 |
|  |  |  |  |  |  |  | (0.334) |
|  |  |  |  |  |  |  |  |
| party_bigSDP |  |  |  |  |  |  | 0.224 |
|  |  |  |  |  |  |  | (0.302) |
|  |  |  |  |  |  |  |  |
| party_bigKESK |  |  |  |  |  |  | -0.077 |
|  |  |  |  |  |  |  | (0.288) |
|  |  |  |  |  |  |  |  |
| party_bigOther |  |  |  |  |  |  | 0.627^*^ |
|  |  |  |  |  |  |  | (0.270) |
|  |  |  |  |  |  |  |  |
| treatmentScientific:party_bigPS |  |  |  |  |  |  | -0.114 |
|  |  |  |  |  |  |  | (0.454) |
|  |  |  |  |  |  |  |  |
| treatmentAltruistic:party_bigPS |  |  |  |  |  |  | -0.173 |
|  |  |  |  |  |  |  | (0.479) |
|  |  |  |  |  |  |  |  |
| treatmentScientific:party_bigSDP |  |  |  |  |  |  | 0.029 |
|  |  |  |  |  |  |  | (0.407) |
|  |  |  |  |  |  |  |  |
| treatmentAltruistic:party_bigSDP |  |  |  |  |  |  | 0.136 |
|  |  |  |  |  |  |  | (0.418) |
|  |  |  |  |  |  |  |  |
| treatmentScientific:party_bigKESK |  |  |  |  |  |  | -0.412 |
|  |  |  |  |  |  |  | (0.403) |
|  |  |  |  |  |  |  |  |
| treatmentAltruistic:party_bigKESK |  |  |  |  |  |  | -0.213 |
|  |  |  |  |  |  |  | (0.408) |
|  |  |  |  |  |  |  |  |
| treatmentScientific:party_bigOther |  |  |  |  |  |  | -0.209 |
|  |  |  |  |  |  |  | (0.367) |
|  |  |  |  |  |  |  |  |
| treatmentAltruistic:party_bigOther |  |  |  |  |  |  | -0.254 |
|  |  |  |  |  |  |  | (0.385) |
|  |  |  |  |  |  |  |  |
| Constant | -2.850^***^ | -2.831^***^ | -2.840^***^ | -2.671^***^ | -2.900^***^ | -2.680^***^ | -2.954^***^ |
|  | (0.113) | (0.091) | (0.091) | (0.145) | (0.097) | (0.156) | (0.224) |
|  |  |  |  |  |  |  |  |
|  | | | | | | | |
| Observations | 7,393 | 7,328 | 7,397 | 7,397 | 7,397 | 7,127 | 7,397 |
| Log Likelihood | -1,638.648 | -1,620.647 | -1,638.251 | -1,636.946 | -1,631.171 | -1,538.328 | -1,628.344 |
| Akaike Inf. Crit. | 3,289.297 | 3,253.293 | 3,294.502 | 3,291.892 | 3,280.342 | 3,088.657 | 3,286.688 |
|  | | | | | | | |
| *Note:* | *p<0.05; **p<0.01; ***p<0.001 | | | | | | |


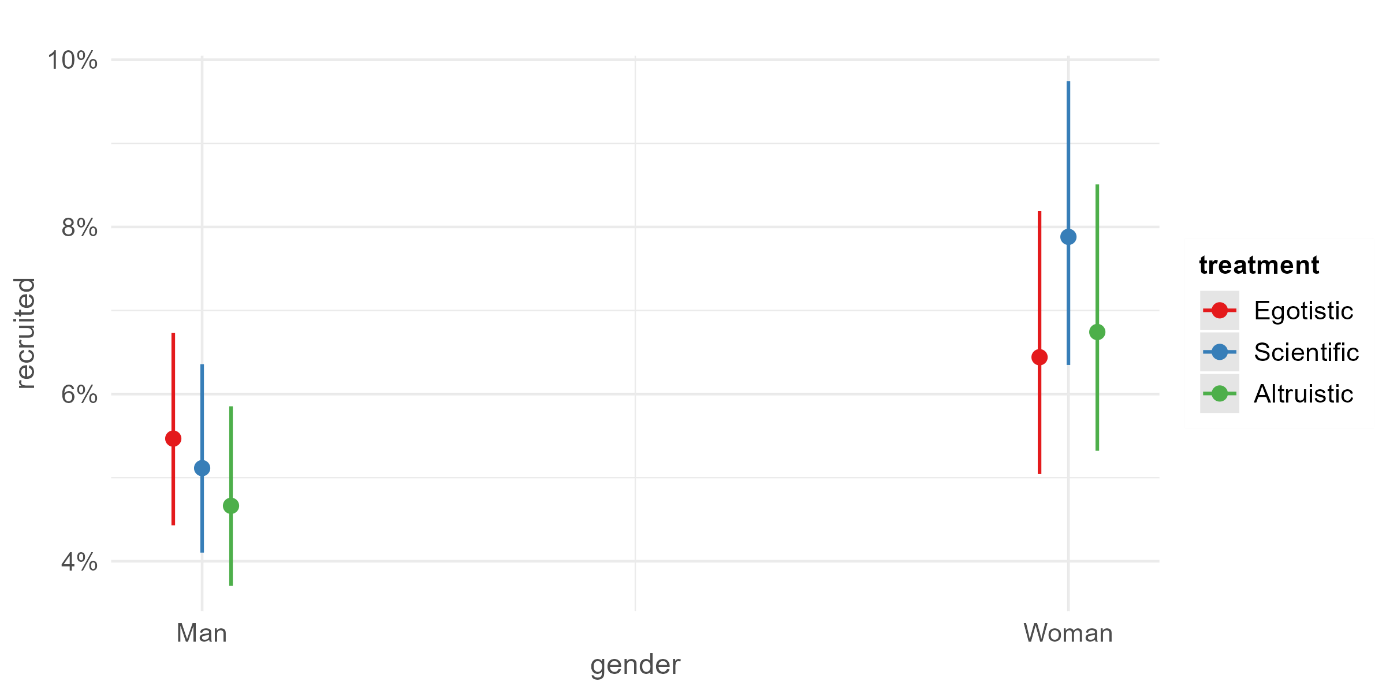


**Figure B2.** Interaction gender and treatment


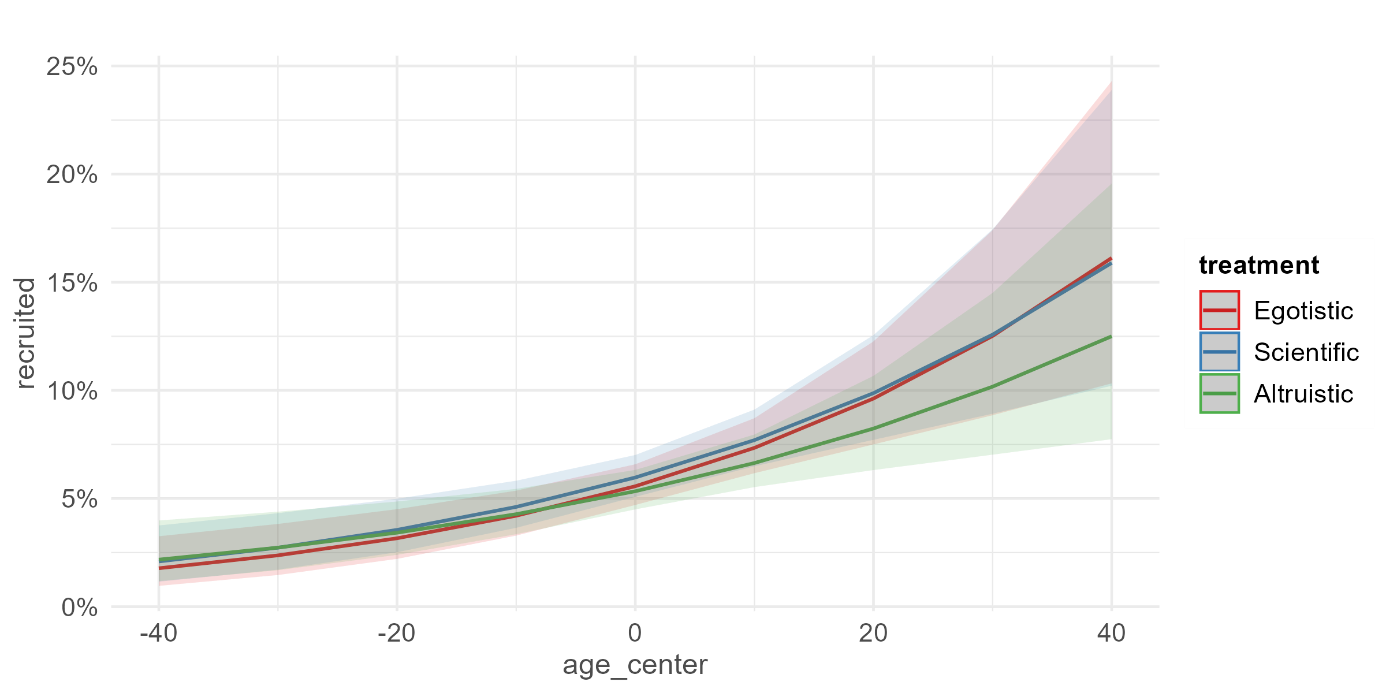


**Figure B3.** Interaction age and treatment


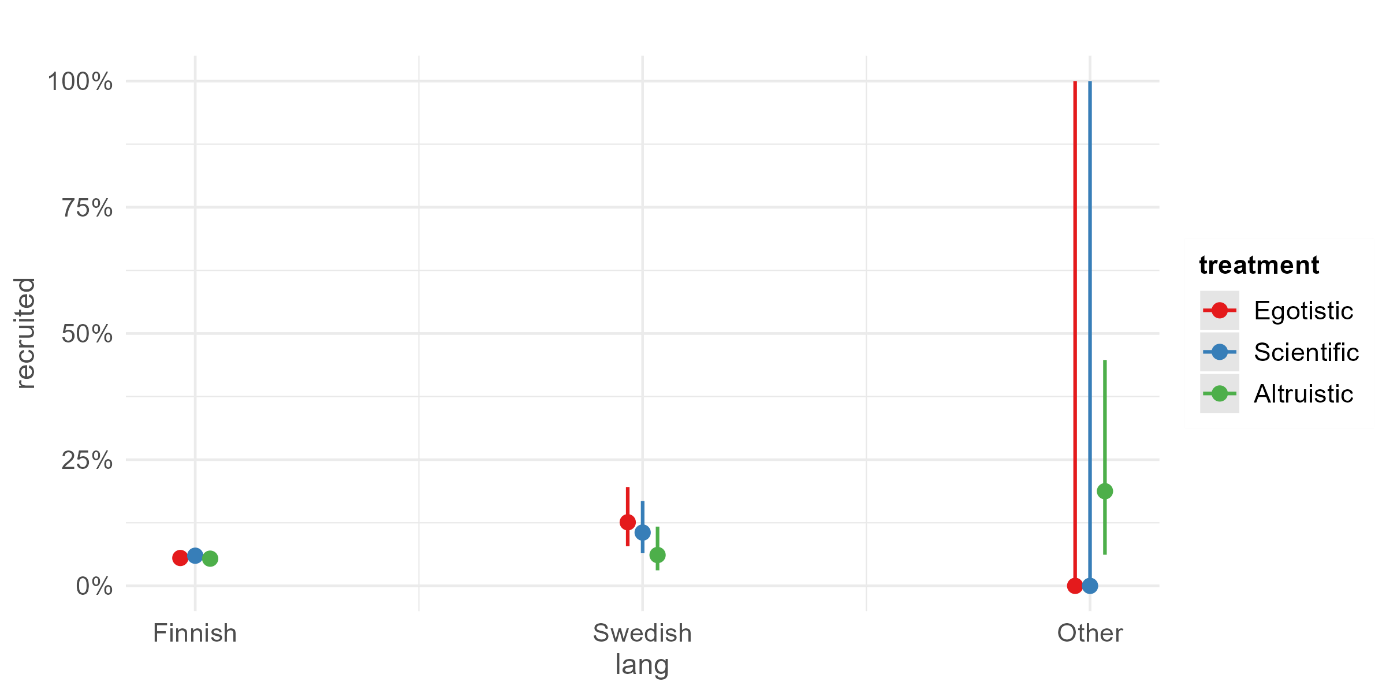


**Figure B4.** Interaction language and treatment


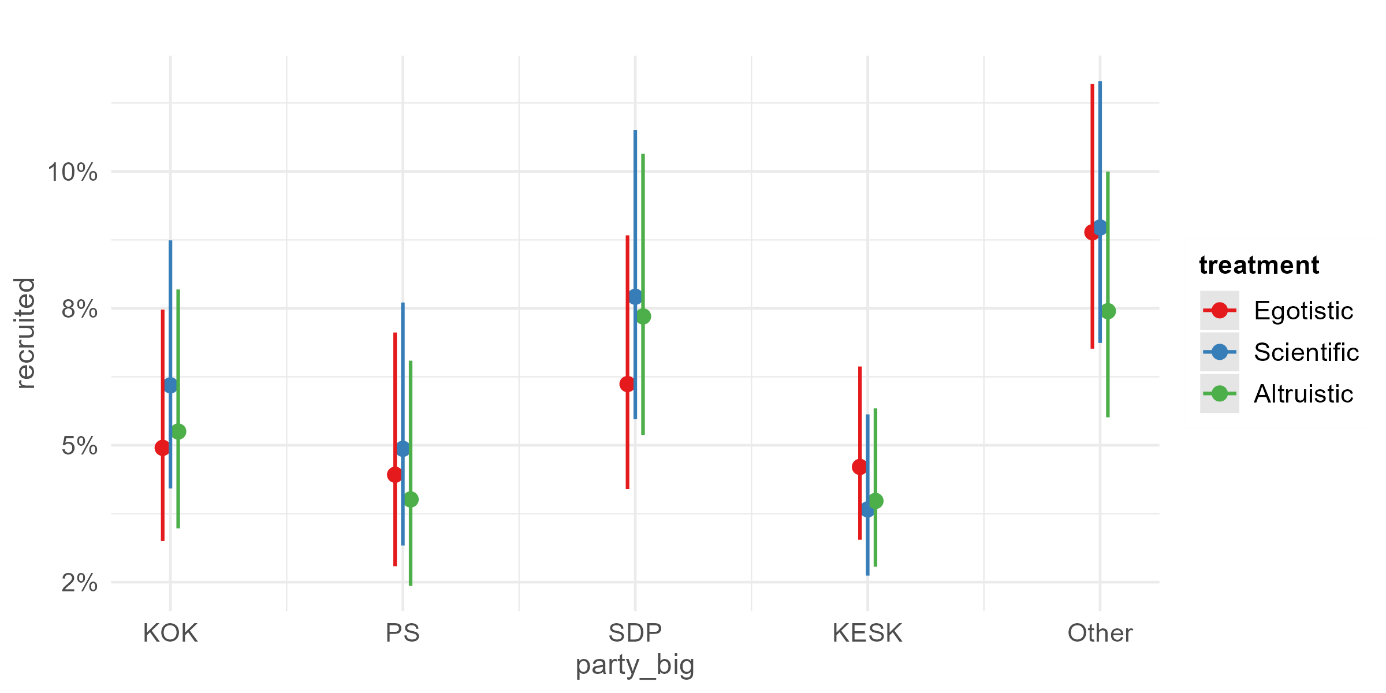


**Figure B5.** Interaction party type big and treatment
