# Supplementary material for: Comparison of altruistic, egoistic, and scientific appeals in the recruitment of politicians to a survey panel
Source: PLoS One. 2026 May 28;21(5):e0349116. doi: 10.1371/journal.pone.0349116 (PMC13218533; doi:10.1371/journal.pone.0349116)
Supplement: Attachment — Submitted filename: Response to reviewers.docx [file pone.0349116.s003.docx]

We thank for the opportunity to revise and resubmit our manuscript. We really appreciated all the comments and have done our best to address them. Below, we respond to each of them in the order as they appear in the decision letter. We look forward to hearing your views on the revised manuscript.

It should be noted that we have rerun all analyses, and excluded the previous party name variable, and for the language variable, merged those with Swedish and other mother tongues. This is done to ensure anonymity for the individuals in the data, and enable the sharing of the data and analysis code.

**REVIEWER 1:**

**Reframe the contribution more explicitly as a methodological demonstration that simple appeal framing does not increase recruitment among politicians, rather than as a theoretical advance in leverage–saliency theory.**

Yes, we agree that this is the right choice for this paper. We are happy to see that the reviewers have understood and appreciated the aim of our paper, which is to contribute to the methodology of recruiting politicians and where the focus is very pragmatic rather than theory-heavy. In the Introduction, we now write as follows, in order to explain the pragmatic aim of the study:

*We study the impact of invitation framing for pragmatic reasons. In their role as elected policymakers who serve the interests of the public, accepting – or expecting – money for advancing research on democracy and perhaps their own political accountability does not seem suitable. This is particularly the case in many countries, such as Finland, where research is primarily publicly funded. Instead of monetary incentives, which may be feasible for attracting ordinary citizens to participate in surveys but not equally appropriate when approaching democratically elected politicians, it is necessary to investigate other ways to improve recruitment success, such as whether different framings could affect politicians’ willingness to participate in a survey panel. We examine the possibility that politicians could be persuaded by appealing to their altruistic sense of responsibility to contribute to research about how democracy works due to their position as elected representatives of the people. Alternatively, a more egoistic appeal suggests that by becoming a panel participant, they are offered access to key findings from the panel, which are highly relevant to their work in local politics. If effective, simple appeals to participate would be an easy and cost-free method for survey researchers to increase cooperation rates in politician samples.*

**Clarify the theoretical rationale for using altruistic, egoistic, and scientific appeals with elite respondents. More strongly justify why politicians, unlike citizens, should be expected to respond differently to these frames.**

This is a fair point. Although maybe arguing for a difference between politicians and citizens is not the main point of the analysis (given that we make no such comparison), we acknowledge that we had not motivated the rationale for using the appeals very well. Admittedly, if there was no reason to think that politicians and citizens would in way react differently, there perhaps would not be any reason to focus specifically on politicians.

We added the following to the beginning of the section that explains the use of the different appeals. As we argue, politicians are likely to have a stronger sense of civic duty, which could make them more likely to react to the appeals.

*The practical issue is how can an invitation be tailored so that it has enough saliency for politicians to cooperate? On the one hand, we assume that politicians have a strong sense of civic duty. Altruism and a sense of civic duty are strong predictors of political engagement (9,10) and local-level politicians are extraordinarily highly engaged individuals. Additionally, research has shown that a motivating factor in becoming a politician in the first place is civic duty, i.e. contributing (altruistically) to society (11). On the other hand, politicians compete for attention to survive in electoral competition. Consequently, it also seems plausible that politicians’ reactions to survey invitations could be affected by more selfish considerations about how to make themselves heard, rather than simply be considered an anonymous data entry (see also 5).*

**Deepen the discussion of null results by considering alternative explanations, such as institutional trust, civic duty, or elite-specific norms, rather than attributing the outcome primarily to information overload.**

As also Reviewer #2 suggested, alternative explanations could still be added. In addition to information overload and weakness of the treatment(s), in this revised version we also discuss sense of civic duty, attitudes towards science and the impact of political context as plausible reasons for why we did not find effects beyond some sub-groups.

**Present subgroup analyses as exploratory rather than confirmatory. This will prevent over-interpretation of interaction effects that lack strong theoretical grounding.**

We agree with the reviewer that especially some of our heterogeneous treatment effect analyses should be viewed as exploratory. We now elaborate on this in the methods section and also conduct descriptive analyses as a complement to the interactions.

*See the Appendix for a closer description of the included variables. Our rationale for including the variables is to improve precision in identifying treatment effects, detect potential biases, and enable us to study heterogeneous treatment effects. As mentioned when discussing the hypotheses, we believe party type affiliation plays a role in the effectiveness of the treatments. Regarding other individual-level demographic variables, we include age due to it regularly being an important predictor of (non-)response in surveys (Lundmark & Backström, 2025). Gender and language are relevant due to potential differences in treatment effects, due to perceptions of being in the majority or minority locally or nationally. Women are generally underrepresented in politics, meaning they may perceive the treatments differently from men, and likewise, the effect of the treatments may differ for those with a mother tongue other than Finnish. The context-level variables, while potentially suffering from ecological validity issues, may also provide insights into the context in which the politicians operate and potential differences in the effects of the treatments. There may be differences in resources and time budgets between municipalities of differing urbanicity, while a bilingual context can also have implications for representativeness, e.g., due to differences in political culture. Still, we view our investigation of these potential heterogenous effects as purely explorative.*

*[...]*

*We also examine heterogeneous treatment effects by comparing recruitment rates across different subpopulations. See Figure A1 and Table A3 in the Appendix. We can note some general trends where women, older, and leftist politicians in general are more prone to join the panel. However, there are no significant differences between treatments within the different groups. Still, while marginally insignificant, the egotistic treatment performed somewhat better among politicians in suburban municipalities. Likewise, the egotistic treatment performed somewhat better, although not statistically significant, among politicians in Swedish-majority bilingual municipalities.*

**Discuss the implications of low response rates more directly, including risks of cumulative attrition in panel studies and limits to generalizability beyond the Finnish context.**

Cumulative attrition, i.e. attrition in a panel over the course of several waves, is indeed a key issue for any panel, along with low response rates. In this paper, we have mentioned low response rates as one reason why successful recruitment is so important. However, we are hesitant about entering the discussion on response rates and cumulative attrition, because we view them as adjacent questions and we do not seek a contribution to the (large) bodies of literature on these subjects.

Nevertheless, generalizability is always an issue with single-country studies. We suggest adding the following to the end of the Discussion:

*While the findings in this analysis are restricted to a single country, the general disposition of politicians in terms of civic duty, patterns of ideological positioning towards science and risk of informational overload, are likely to be similar across other Western democracies. However, the varying political contexts may generate differences, which diminish the generalizability of the findings.*

**Improve readability by streamlining descriptive material and moving some tables and statistical outputs to appendices.**

We understand that tables and figures may affect the readability of an article. At this point, we suggest moving Table 1 to the Appendix, which we have done. It is now Table A3. If the reviewer or the editor feels more removals are needed, Figures 4 and 5 could be included in the Appendix as well, because they are somewhat explorative. Nevertheless, we felt they belong to the storyline of the paper and for the time being kept them in the body text.

**REVIEWER 2:**

**The assumption that an egoistic appeal works because politicians value access to the panel’s findings is strong and should be treated more cautiously. A growing body of elite-survey research suggests that many politicians do not perceive direct benefits from public-opinion information, nor do they believe such information meaningfully advance their careers. Acknowledging this in the discussion section would help readers understand why the treatment may not have produced significant differences (i.e., perhaps because the stimulus was not strong enough to generate a behavioral response).**

It is also our interpretation that among some other likely reasons, the treatment was not strong enough to provoke a reaction. We agree with the reviewer that suggesting that the egoistic appeal would work due to politicians valuing access to the panel’s findings sounds unlikely. In the initial submission, we wrote this in one sentence in the introduction, where we explained the rationale behind the treatments. Now, based on various reviewer comments, we have instead emphasized the actual treatment text, which read “By participating in the panel, you will be able to voice your opinion about democracy in Finnish municipalities and about different societal issues.” Thus, the expected impact would be due to politicians’ desire to make their own opinions heard, not access to the panel’s findings. Moreover, the reviewer is correct in pointing out that politicians likely do not consider survey data as being very useful or relevant to their work. In our analysis, this is probably best reflected in the fact the scientific altruistic message was mostly ineffective.

We made two changes. First, we removed the sentence suggesting that politicians would participate because of access to the panel’s findings. Second, we agree with the reviewer that it would be useful to comment in the Discussion on politicians’ attitudes toward opinion data, as one possible reason for the lack of treatment effects. With a reference to Walgrave & Soontjens (2023), we suggest the following addition:

*Politicians do not see surveys as particularly useful sources of information when it comes to public opinion, but instead prefer other sources, such as direct citizen contacts (22). The hesitancy about surveys and polls in general could also help explain the relative ineffectiveness of the scientific benefit message and the low overall cooperation rate.*

**Different forms of egoism and altruism may appeal to different types of politicians, depending on institutional, electoral, and media contexts. It would be useful for the authors to acknowledge that such cross-context and role-based variation could partly explain the absence of clear treatment effects.**

This is a very good point. We added the following to the Discussion:

*Moreover, also the political context of politicians differs significantly, plausibly affecting how they react to survey invitations. Being in a position of power versus in political opposition could affect their sense of altruism and selfishness and, consequently, their willingness to engage in a survey panel. Those in power might have even less capacity to devote to anything outside the political agenda, while those who are relatively powerless might have more time and even a stronger need to be able to voice their opinions any way they can – even through a survey. In sum, different appeals could work depending on the political context and in this analysis, we were unable to account for the variation in municipal-level political dynamics.*

**The paper refers to three experimental groups repeatedly before clearly specifying what these groups are. Introducing the treatment conditions earlier in the paper would provide an overview of the design to the reader and make the design easier to follow.**

This is a good point and simple to fix. We now explain the treatments already in the introduction where they are mentioned for the first time.

**The second hypothesis is under-theorized relative to the first and third. Strengthening the theoretical justification here, perhaps by linking it to distinct mechanisms or prior findings, would improve coherence across the hypotheses. There was a brief mention about left-wing politics, but I haven't seen much about past research detailing the mechanism behind this.**

We readily admit that H2 was too superficially justified. We rewrote the justification, with reference to https://doi-org.ezproxy.utu.fi:2443/10.1177/07311214211022391 and http://dx.doi.org/10.1146/annurev-soc-030320-035037. Including new references to previous research, it now reads as follows:

*In addition to a general altruistic appeal, we include a more specific version called the scientific appeal. It emphasizes the importance of volunteering as a panel member to contribute to the advancement of science. In line with the saliency theory, the scientific treatment message expects to increase the sense of relevance of participating, which could be particularly effective among politicians who supposedly wish to contribute to the common good. However, the saliency of scientific benefits of participating could plausibly depend on ideological leaning. In recent years, climate change and Covid-19 have become strongly contentious issues in terms of attitudes towards science and conservatives display significantly less trust in science than liberals and/or left-leaners (16–18). Distrust in science is also associated with behavioral outcomes, such as lower support for science-based policy and for scientifically grounded recommendations (16). Consequently, politicians in the political left could more likely be persuaded by the scientific appeal, because they tend to have more faith in science and also behave accordingly.*

**I was unclear about the pre-registration process. The text refers to a pre-registration (“see the pre-registration for more details”) but then suggests that it was shared only with the editors. I was not able to identify a separate submission document that details the pre-registration process. Reviewers know who the authors are, so I don't understand why the authors preferred not to reveal it. If the document is really available only to the editors, it prevents reviewers from assessing deviations from the original design, which undermines the purpose of pre-registration. Clarifying this and, ideally, sharing the pre-registration document as supplementary material would increase transparency and credibility.**

We had somehow simply missed that the reviewers will get access to our names already at this point and therefore retracted the link to the pre-registration from the manuscript, which is of course the praxis with most journals. The link is <https://doi.org/10.17605/OSF.IO/C3T4X> and it is now also included in the manuscript. Our apologies for the confusion.

**With over 7,000 politicians in the sample, the study is large enough to examine heterogeneous treatment effects. It would strengthen the contribution to explore whether the treatment effects differ across subgroups. Even descriptive exploration of heterogeneity would enrich the interpretation of otherwise null or weak average effects.**

We agree that heterogeneity is worth studying, considering the sample size. While we already did this in our previous version of the paper, when looking at interactions, we have now made this more explicit in the text, and conducted further analyses.

At the end of the introduction we now closely touch upon our findings:

*There are no statistically significant differences across the different appeals. However, we find that women, older, leftist, representing a bilingual or urban municipality are more likely to comply with the panel invitation than their counterparts. Still, we find some heterogeneous effects: egotistic appeals are more effective for politicians representing suburban municipalities, and altruistic appeals are less effective for politicians representing bilingual municipalities with a Swedish majority. While we cannot rule out the possibility that other types of appeals can be more effective, we interpret the findings as suggesting that researchers need to look beyond the framing of invitations in order to maximize recruitment rates among politicians. The perceived trustworthiness and reputation of the inviting organization are potentially more important factors behind politicians’ willingness to commit to a survey (panel).*

After Figure 1, we now also mention:

*We also examine heterogeneous treatment effects by comparing recruitment rates across different subpopulations. See Figure A1 and Table A3 in the Appendix. We can note some general trends where women, older, and leftist politicians in general are more prone to join the panel. However, there are no significant differences between treatments within the different groups. Still, while marginally insignificant, the egotistic treatment performed somewhat better among politicians in suburban municipalities. Likewise, the egotistic treatment performed somewhat better, although not statistically significant, among politicians in Swedish-majority bilingual municipalities.*

**The discussion of low acceptance rates across treatment groups suggests that bias from rare events could affect the estimates. Replicating the main models using a penalized maximum-likelihood estimator, such as Firth’s logistic regression for rare events (see King & Zeng; Allison), would be a valuable robustness check.**

We thank the reviewer for the suggestion. It is true that regular MLE regressions can be biased when dealing with unbalanced data. We have now run Firth’s logistic regression as a robustness check (Appendix Table A6). We find no substantive differences between the two models and decide to continue using the MLE regression. We elaborate on this and other model quality measures in the paper:

*See Appendix Table A5 and Table A6 for different models used to predict recruitment and the models’ fit. The chosen model (model 4) had the best fit, and we further examined model quality in Figure A2. We conclude that the model had a good fit, although it showed some issues with binned residuals, suggesting a slight risk of overpredicting recruitment for some groups. Across all four models, the overall treatment effects are not significant. As a robustness check, we also tried running the same model using Firth’s logistic regression, which can be more suitable when working with unbalanced data (King & Zeng, 2001). However, we note no substantive differences between the regular MLE logistic regression and Firth’s logistic regression (Table A7 in the Appendix), meaning we continue using the MLE logistic regression model.*

**The manuscript lacks a clear methodological section explaining why specific estimators were chosen and how balance was assessed. Most experimental studies report randomization checks or balance tests; this paper does not clearly do so. Table A1 includes significance tests, but it is unclear what they really tested, and many are statistically significant. A simple regression predicting treatment assignment with covariates would demonstrate whether randomization succeeded. There are many other tables in the appendix, but it is difficult to understand what they do. A detailed discussion accompanying those tables would be very helpful. The authors should walk readers through these diagnostics explicitly.**

We thank the reviewer for pointing out the current lack of a randomization check. We have now added this to the appendix (Table A1), where we look at how the distribution between treatment groups overall. We confirm that there are no statistically significant differences between treatment groups in the variables of interest (gender, age, language, municipality type, municipality language ratio, political party classification, and political party), meaning the randomization was successful, due to there being no statistically significant differences. We have added a footnote to the paper when discussing the treatments:

*See Appendix Table A1 for a randomization check. We do not find any statistically significant differences between the treatment groups at the point of treatment assignment.*

We have also cleaned up the appendix and now discuss the included tables and robustness checks in more detail in the actual paper. We now also only have one single Appendix A for ease of readability. Also, we now motivate further which variables we include in our analyses, namely by adding this text at the end of the Material and methods chapter:

*See the Appendix for a closer description of the included variables. Our rationale for including the variables is to improve precision in identifying treatment effects, detect potential biases, and enable us to study heterogeneous treatment effects. As mentioned when discussing the hypotheses, we believe party type affiliation plays a role in the effectiveness of the treatments. Regarding other individual-level demographic variables, we include age due to it regularly being an important predictor of (non-)response in surveys (Lundmark & Backström, 2025). Gender and language are relevant due to potential differences in treatment effects, due to perceptions of being in the majority or minority locally or nationally. Women are generally underrepresented in politics, meaning they may perceive the treatments differently from men, and likewise, the effect of the treatments may differ for those with a mother tongue other than Finnish. The context-level variables, while potentially suffering from ecological validity issues, may also provide insights into the context in which the politicians operate and potential differences in the effects of the treatments. There may be differences in resources and time budgets between municipalities of differing urbanicity, while a bilingual context can also have implications for representativeness, e.g., due to differences in political culture. Still, we view our investigation of these potential heterogenous effects as purely explorative.*

**Assuming randomization was successful, control variables may not be necessary. Presenting models without controls would help assess the robustness of the findings and avoid potential post-treatment bias. In other words, comparing simple and full specifications would increase confidence in the results.**

We agree that it is also important to show more parsimonious models. We do so now in Table A6, where we show the four different models. We can see that the overall treatment effects are not statistically significant across all models. This is referenced also in the paper now when discussing the choice of model and model fit.
