# Supplementary material for: Comparison of altruistic, egoistic, and scientific appeals in the recruitment of politicians to a survey panel
Source: PLoS One. 2026 May 28;21(5):e0349116. doi: 10.1371/journal.pone.0349116 (PMC13218533; doi:10.1371/journal.pone.0349116)
Supplement: Attachment — Submitted filename: Response letter_R2.docx [file pone.0349116.s004.docx]

**Response letter**

Dear editor and reviewers

Thank you for the opportunity to review and resubmit our paper “Comparison of altruistic, egoistic, and scientific appeals in the recruitment of politicians to a survey panel”.

We have now revised the paper and have submitted a version both with and without tracked changes. Also, we have now submitted replication data and code, as well as figures in TIFF format (300 dpi).

Here follows a detailed response to Reviewer 1’s concerns.

1. Strengthen the theoretical justification for the three treatments

**Response:** The reviewer would still like to see a better theoretical discussion of the mechanism behind the rationale for the treatments. We have added social conformity theory as the fundamental explanation for why we expect that politicians could respond to our treatments.

In addition to some smaller changes elsewhere, the theory section now includes the following:

*The practical issue is how can an invitation be tailored so that it has enough saliency for politicians to cooperate? Fundamentally, we draw on the central tenet of social conformity theory, according to which people’s behaviors and actions depend on what they assume is expected of them because of their social positions and role in any situation (see e.g. ,9,10). Thus, performing in their role as an elected politician when they receive the invitation, we expect the politicians to be persuaded by appeals that refer to what is perhaps societally expected behavior in this particular situation. On that note, we assume that politicians may feel that demonstrating have a strong sense of civic duty is something that conforms to social expectations. Additionally, research has shown that altruism and a sense of civic duty are strong predictors of political engagement (11,12) and local-level politicians are extraordinarily highly engaged individuals. Additionally, research has also shown that a motivating factor in becoming a politician in the first place is civic duty, i.e. contributing (altruistically) to society (13).*

2. Make it clearer that the subgroup analyses are exploratory

**Response:** Throughout the paper, we have eased up the wording regarding the heterogeneous analyses, making it clearer that they should be viewed as exploratory and that their statistical power is more limited.

Namely:

*In Figures 4 and 5, we highlight statistically significant findings outside of the hypotheses simply as an exploratory approach to identify potential underlying effects that are otherwise hidden by the main effects.*

*[…]*

*Against the hypotheses, the findings suggest that, besides some potential effects among certain sub-populations, different invitation appeals do not affect their propensity to accept the invitation.*

*[…]*

*The interactions nevertheless revealed some potential effects*

*[…]*

*Still, these subgroup analyses should be interpreted with caution, as statistical support is weaker for smaller groups, and successful recruitment, to begin with, was quite rare.*

3. Discuss limitations regarding generalizability, both regarding the Finnish and local-level cases.

**Response:** We have now elaborated in the final paragraph of the conclusions regarding this:

*While the findings in this analysis are restricted to a single country and at the local political level*

*[…]*

*Future research should strive to replicate the experiment both in similar and different contexts. Replicating the experiment in a similar context would provide insight into whether the null findings are de facto the result of nonexistent effects or simply a Type II error. Replication in a very different political context would give insight into whether the findings are generalizable also outside of the quite distinct Finnish local-level political system.*

4. Move methodological considerations into the main text

**Response:** We have now deleted the two footnotes on randomization checks and model selection and have instead included more detailed descriptions in the main text.

When discussing the randomization, we have added:

*As a randomization check, we compare the sample compositions of the three treatment groups concerning gender, age, language, municipality type, municipality language ratio, and political party classification. We find no statistically significant differences between the groups, indicating successful randomization (see Table A1).*

When discussing the predicted probabilities, we have added:

*We also use logistic regression and predicted probabilities to examine predictors of recruitment. We test four different models and compare their fit (see Appendix Table A5 and Table A6. The chosen model (model 4) had the best fit, and we further examined its quality using the performance-package (Lüdecke et al., 2019). We conclude that the model had a good fit, although it showed some issues with binned residuals, suggesting a slight risk of overpredicting recruitment for some groups (see Figure A2). Across all four models, the overall treatment effects are not significant. As a robustness check, we also tried running the same model using Firth’s logistic regression, which can be more suitable when working with unbalanced data (20). However, we note no substantive differences between the regular MLE logistic regression and Firth’s logistic regression (Table A7 in the Appendix), meaning we continue using the MLE logistic regression model.*
