# Supplementary material for: Comparison of altruistic, egoistic, and scientific appeals in the recruitment of politicians to a survey panel
Source: PLoS One. 2026 May 28;21(5):e0349116. doi: 10.1371/journal.pone.0349116 (PMC13218533; doi:10.1371/journal.pone.0349116)
Supplement: Attachment — Submitted filename: Response to reviewers_R3.docx [file pone.0349116.s005.docx]

Thank you for the review comments and for the opportunity to revise and resubmit once more. Below, we respond to the remaining comments in the order as they appear in the decision letter. We look forward to hearing what you think of the revised version of our manuscript.

1) The theoretical contribution would benefit from a more explicit articulation of the mechanisms connecting role-based expectations to the specific experimental manipulations.

While the reviewer appreciated the revised theoretical section, something seems to still be lacking in the way it connects with the experimental design. Although the we and the reviewer may have slightly different understandings of what constitutes a ‘mechanism’ in this type of research, we have done our best to articulate even more explicitly what effects we expected from the design. As we understand it, a ‘mechanism’ in this context can only be a reaction in the respondent’s mind, prompted by words in the actual panel invitation. To some extent, our design is exploratory, because we simply do not know what kind of an appeal is going to work best. Hence, we expect the same basic ‘mechanism’ to be at work in all instances, which is very simply that appealing to a person’s sense of duty will make them more likely to comply with a request. To be very clear on this, we added the following:

Regarding the altruistic treatments: Therefore, the mechanism, which we expect to trigger a response in the respondents as they read the invitation with the altruistic appeals, is the feeling that politicians’ are expected to display a sense of duty towards the good of society.

Regarding the egoistic treatment: In this case, the mechanism assumed to be at play is the more self-centered need to seek visibility and focus, rather than the need to conform to duty-based role expectations.

2) Parts of the discussion still attribute substantive meaning to marginal or statistically weak patterns. I would encourage further restraint here, ensuring that these findings are presented strictly as hypothesis-generating.

We understand that the reviewer wants us to be even more cautious about the interpretation of the findings than what we currently are. In our own reading of how we present the findings, we mostly just say that the appeals we used did not seem to work, rather than make strong claims about statistically weak connections (if this is what the reviewer means). For example, we begin the discussion by referring to ‘potential effects among some sub-populations’. Additionally, we conclude that ‘Still, these subgroup analyses should be interpreted with caution, as statistical support is weaker for smaller groups, and successful recruitment, to begin with, was rare’. These conclusions, in our view, do not attribute significant substantive meaning to the findings. However, we have revisited this section once more and made small adjustments, which further emphasize the uncertainty of the findings. This also includes our response to the next reviewer comment.

3) Given the very low baseline participation rate, it is plausible that behavioral variation is constrained, limiting the observable impact of treatments. This alternative explanation limited elasticity rather than absence of effect should be more directly incorporated into the interpretation.

The reviewer makes a valid and useful point with this comment, which we see as related to the previous one. We added the following to the discussion where we comment on the null findings:

*Second, due to a relatively low acceptance rate, it could be that the experiment did not capture enough behavioral variation among politicians to fully reveal the impact of the treatments.*

4) The discussion introduces several plausible mechanisms, such as information overload, institutional trust, and reputational considerations, but these remain post hoc explanations rather than analytically integrated alternatives. The manuscript would be stronger if it more explicitly positioned these mechanisms alongside the tested framework, even if only as directions for future research.

Our aim has always been to present these as plausible mechanisms to be tested by future research, rather than ‘post hoc explanations’. After all, we cannot address these possibilities empirically with our (pre-registered) design anyway, so they cannot really be anything else but directions for subsequent analyses. With small adjustments in the discussion, we have now framed them more clearly as precisely that – hopefully to avoid any further confusion.
